# Forest Soil Fungal Community Elevational Distribution Pattern and Their Ecological Assembly Processes

**DOI:** 10.3389/fmicb.2019.02226

**Published:** 2019-10-04

**Authors:** Yuyu Sheng, Wei Cong, Linsen Yang, Qiang Liu, Yuguang Zhang

**Affiliations:** ^1^Research Institute of Forest Ecology, Environment and Protection, Chinese Academy of Forestry, and the Key Laboratory of Biological Conservation of National Forestry and Grassland Administration, Beijing, China; ^2^Shennongjia National Park Administration, and Hubei Provincial Key Laboratory on Conservation Biology of the Shennongjia Golden Monkey, Shennongjia, China

**Keywords:** soil fungal diversity, community assembly processes, elevational gradient, environmental factors, Illumina MiSeq sequencing

## Abstract

Soil fungi play vital roles in natural ecosystems, however, their community distribution patterns along different environmental gradients and ecological assembly processes remain unclear. In this study, Illumina MiSeq sequencing was used to investigate the soil fungal community structures of five different forest types along an elevational gradient, and a framework based on a null model was adopted to quantify the relative contribution of deterministic and stochastic ecological assembly processes. The results showed that the majority of soil fungal OTUs were derived from Zygomycota, Basidiomycota, and Ascomycota. Soil fungal community structure differed significantly among the five sites (*P* < 0.01), and the fungal α-diversity decreased as elevation increased (*P* < 0.01). The null model showed that the relative contribution of stochastic processes (37.78–73.33%) was higher than that of deterministic processes (26.67–62.22%) within the same forest type, while that of deterministic processes (35.00–93.00%) was higher than stochastic processes (7.00–65.00%) between forest types. These results suggest that forest soil fungal diversity decreased significantly with increasing elevation, and that deterministic processes may be key factors influencing soil fungal community assemblies among forest types. The results of this study provide new insight into soil fungal distribution patterns and community assembly processes in natural forest ecosystems.

## Introduction

Soil microbial community structures and diversity along different environmental gradients have received a great deal of attention in recent years (Zhang et al., 2014; Geml et al., 2017; Liu et al., 2018). In mountain ecosystems, elevational gradients are characterized by environmental changes in climate, vegetation, and soil properties (Siles and Margesin, 2016; Liu et al., 2018). Examination of these changes has facilitated the development of basic ecological theory and predictions of consequences of climate change (Bryant et al., 2008; Zhang et al., 2014). However, the majority of studies conducted to date have focused on plants, animals (Lomolino, 2010; Mccain, 2010; Cong et al., 2013), or soil bacteria (Bryant et al., 2008; Yang et al., 2014; Zhang et al., 2014; Liu et al., 2017).

To date, varying soil fungal α-diversity (species richness or Shannon indices) distribution patterns along elevational gradients have included monotonous decrease (Schinner and Gstraunthaler, 1981), a humpback pattern (Wang et al., 2015), or no significant pattern (Coince et al., 2014; Siles and Margesin, 2016; Liu et al., 2018). These findings indicate that soil fungal elevational distribution patterns are complex in natural ecosystems. Both biotic and abiotic conditions change significantly along elevation gradients (Geml et al., 2017). Numerous studies have used plant diversity as a predictor of soil microbial diversity (Kardol and Wardle, 2010; Hiiesalu et al., 2014; Prober et al., 2015). However, there is increasing evidence that soil microbes may significantly drive plant diversity and community composition (Prober et al., 2015; Teste et al., 2017). Therefore, although there is theoretical support for linkages between plant and soil microbial diversity, empirical evidence is inconclusive and somewhat uncoupled from alpha to beta diversity (Wardle, 2006; Millard and Singh, 2010; Gao et al., 2013; Prober et al., 2015).

Understanding ecological assembly processes of soil fungal communities is a significant challenge (Stegen et al., 2012). In general, ecological assembly processes are classified into deterministic and stochastic processes (Chase and Myers, 2011; Stegen et al., 2012, 2013; Yang et al., 2017), with four potential sub-processes - selection, dispersal, drift, and speciation (Vellend, 2010). Selection is a deterministic process induced by abiotic environmental change and microbial interactions (Stegen et al., 2012). Dispersal, drift, and speciation are more likely to be stochastic processes (Chase and Myers, 2011). Emerging evidence suggests that microbial communities are simultaneously structured by both deterministic and stochastic processes (Ofiteru et al., 2010; Stegen et al., 2012; Valverde et al., 2014; Moroenyane et al., 2016). Morrison-Whittle and Goddard (2015) found that the average niche contribution (selective effect) was approximately four times that of geographic location. Valverde et al. (2014) suggested that desiccation plays an important role in the formation of bacterial communities and that the selection process is more important in wet than in dry soil. However, these studies did not consider the assembly processes of selection, dispersal, drift, or speciation. Therefore, our knowledge regarding the ecological processes driving soil fungal composition patterns is limited.

In this study, we used the Illumina MiSeq platform to sequence soil fungal communities in five different forest types along an elevational gradient of 1000–2800 m on Shennongjia Mountain, one of the most species-rich areas in China due to its unique geographic location and complex topography (Cong et al., 2015). This mountain contains a variety of forest types extending from 900 m above sea level (masl) to approximately 3000 masl, providing an ideal location for ecologists to study biological elevational distribution patterns (Zhang et al., 2014). The specific goals of the present study were to: (1) determine the soil fungal community composition and distribution patterns along an elevational gradient; (2) determine the ecological assembly processes making important contributions to shaping soil fungal communities; (3) determine which environmental factors are key drivers of soil fungal diversity.

## Materials and Methods

### Site Description and Soil Sampling

The study sites were situated in Shennongjia National Park (SNP), China. The mean annual temperature (MAT) and precipitation (MAP) in the park are 7.2°C and 1500 mm, respectively (Ma et al., 2008). Five forest types were selected along the elevational gradient at SNP, an evergreen broadleaved forest at approximately 1030 m above sea level (EBF1030), a deciduous broadleaved forest at approximately 1780 m above sea level (DBF1780), a mixed coniferous and deciduous broadleaved forest at approximately 2300 m above sea level (MF2300), a coniferous forest at approximately 2550 m above sea level (CF2550), and shrubland at approximately 2750 m above sea level (SL2750). In August 2011, eight to ten study plots (20 m × 20 m) per site were established with approximately 20 m between adjacent plots. At each plot, ten to fifteen soil cores were taken and sieved with 2 mm mesh. Eight or ten samples were collected from each forest type. Soil samples were preserved on ice during transportation and divided into two parts. One part was used to measure soil physicochemical parameters (stored at 4°C) and the other was used as a sample from which to extract DNA from (stored at −80°C).

### Soil Physicochemical Parameters and Plant Diversity Measurement

Soil organic carbon (SOC), total nitrogen (TN), total phosphorus (TP), available nitrogen (AN), available phosphorus (AP), pH, and moisture (Mo) were measured as previously described (Bao, 2000). MAT and MAP were collected from IPCC^1^ (1950–2000). Plant communities including arbor and shrub layers were surveyed and information such as plant species, number, canopy, height, diameter at breast height (1.3 m) for each tree and ground diameter for each shrub were recorded.

### DNA Extraction

Soil microbial DNA was extracted using the E.Z.N.A. Soil DNA Kit (OMEGA Bio-tek, United States) and purified by electrophoresis with a 1% agarose gel. The quality and quantity were determined based on the absorbance at ratios of 260/280 and 260/230 nm, which was measured using a NanoDrop ND-1000 Spectrophotometer (NanoDrop, Wilmington, DE, United States).

### Soil Fungal ITS Illumina Sequencing and Raw Data Processing

For fungal ITS (internal transcribed spacer), an amplicon of 309 bp in ITS2 of the soil fungi ribosome gene was targeted using the following primers: gITS7F (5′-GTGARTCA TCGARTCTTTG-3′) and ITS4R (5′-TCCTCCGCTTATTG ATATGC-3′) (Zhou et al., 2016), which were combined with Illumina adapter sequences and sample-specific barcodes (Bahram et al., 2016). The amplification mix contained 5 μl 10 × Taq Buffer, 1.5 μl dNTP Mixture, 0.5 μl Taq, 2 μl BSA (5 mg/mL), and 1 μl primers. Additionally, 1 μl soil microbial template DNA (∼25 ng/μl) and 38 μl ddH_2_O were added to give a total volume of 50 μl. The thermal cycling conditions were as follows: 94°C for 1 min, followed by 35 cycles of 94°C for 20 s, 56°C for 25 s and 68°C for 45 s, then 10 min at 68°C. Purified PCR products were mixed and quantified to construct a DNA library, after which a MiSeq Reagent Kit (Illumina, San Diego, CA, United States) was used for 2 × 250 bp paired-end sequencing on a MiSeq machine (Illumina, San Diego, CA, United States).

The raw data were preprocessed using a Galaxy pipeline^2^. Raw sequences with barcodes were assigned (with 1.5 maximum number of error) to samples and primers were trimmed with 1.5 mismatches allowed. Forward and reverse fragments with at least 30 bp were combined using FLASH (Magoč and Salzberg, 2011), then trimmed by Btrim with a threshold of QC > 20 over a 5-bp window size (Zhou et al., 2016). Joined sequences <200-bp or containing ambiguous bases (i.e., N) were deleted (Zhou et al., 2016) and the remaining sequences were then trimmed to between 240 and 350 bp. After removing chimeras by UCHIME (Edgar et al., 2011), the operational taxonomic units (OTUs) were classified by UCLUST at a 97% threshold similarity. Finally, sequence numbers were resampled at 10,000 sequences per sample and taxonomic assignment performed using the RDP classifier with 50% confidence (Zhao et al., 2016; Wang et al., 2017).

### Statistical Analysis

Plant diversity was represented using Shannon and richness indices. Soil fungal diversity was represented using the soil fungal ITS Shannon and richness indices determined by Illumina sequencing. The Anosim dissimilarity test (based on the Bray-Cutis and Jaccard distances) was used to calculate fungal community differences. A partial Mantel test was used to determine the correlations of environmental factors with soil fungal diversity. Fungal function guild for ITS was annotated using FUNGuild (Nguyen et al., 2016). All statistical analyses were performed using the R package “vegan” (Oksanen et al., 2016), the Institute for Environmental Genomics online platform^3^, or Galaxy pipeline (see footnote 2) for FUNGuild.

A framework that relied on a null model was used to discern stochastic and deterministic ecological processes and to quantify their relative contributions (Stegen et al., 2012, 2013). This framework relies on the phylogenetic evolutionary distance (Bray-Curtis method) separating OTUs found in one community from those found in a second community (Stegen et al., 2012). Moreover, this framework can identify feature that impose selection or dispersal limitation and quantitatively estimates the effects of selection, drift acting alone and homogenizing dispersal, and dispersal limitation acting alongside drift (Stegen et al., 2013). We first used the abundance-weighted β-mean-nearest taxon distance (βMNTD) to quantify the phylogenetic distance between microbial communities and obtained the observed βMNTD (βMNTDobs) and a null distribution βMNTD (βMNTDnull). A value of | βNTI | > 2 indicates that selection governs the turnover between pairwise microbial communities. The Bray-Curtis-based Raup-Crick (RCbray) index, which is the magnitude of deviation between the observed Bray-Curtis and expected Bray-Curtis values, was introduced when | βNTI | < 2. In the context of | βNTI | < 2, a value of RCbray > +0.95 indicates the observed turnover is governed by Dispersal Limitation, RCbray value <−0.95 indicates the influence of Homogenizing Dispersal, and a | RCbray | value <0.95 indicates the influence of drift. According to the βNTI value between the same or variable sample sites, the number of each ecological process was obtained, and the percentage of each ecological process in the module was manually calculated. Analyses were conducted using “picante” package in R and FileZilla software.

## Results

### Soil Physicochemical and Plant Diversity Parameters

Soil physicochemical properties and plant diversity are presented in Table 1. Significant positive relationships were found between elevation and SOC concentration, elevation and TN concentration, elevation and AN concentration, and elevation and MAP. Significant negative relationships were found between elevation and pH and elevation and MAT. In addition, plant diversity based on the Shannon and richness indices exhibited significant decreases in diversity with increasing elevation (Supplementary Figure S1A).

**TABLE 1:** Soil physicochemical parameters, and soil fungal and plant diversity indices.

**Parameters**	**EBF1030**	**DBF1780**	**MF2300**	**CF2550**	**SL2750**
Altitude (m)	1030.886.90e	1784.712.21d	2316.96.94c	2561.17.50b	2750.85.32a
Number of plots	8	10	10	10	10
Soil organic carbon (g/kg)	52.5810.21a	29.141.02b	60.102.36*a*	60.824.34a	61.823.99a
Total nitrogen (g/kg)	4.140.63a	1.920.11b	4.810.20a	4.290.25a	4.600.30a
Available nitrogen (mg/kg)	286.1933.91b	183.5310.58c	338.3911.93b	342.5824.00b	415.1530.49a
Total phosphorus (g/kg)	1.200.41b	0.230.01c	0.820.03*b*c	0.630.03bc	2.030.34a
Available phosphorus (mg/kg)	6.171.01ab	3.300.11b	9.240.95ab	10.830.13a	8.640.86ab
pH	6.590.32a	5.350.15b	5.290.10b	4.980.04b	4.420.04c
Mean annual temperature (°C)	12.700.00a	9.500.00b	5.801.33c	4.000.00e	4.580.61d
Mean annual mean precipitation (mm)	1067.000.00e	1234.000.00d	1406.805.87c	1479.000.00a	1462.901.99b
Plant Shannon index	2.730.16a	2.140.22b	2.470.10ab	2.150.07b	0.860.07c
The number of plant species	34.000.26a	28.502.32ab	32.100.98a	22.401.36b	5.100.66c
Soil fungal ITS Shannon index	3.640.26a	3.810.23a	3.050.17ab	3.100.18ab	2.510.14b
Soil fungal ITS richness index	532.1360.89ab	752.2077.39a	476.9043.66b	442.3039.96b	327.3030.62b

*Data were presented in mean value ± standard error, different lowercase letters within the same row indicate significant difference at *P* < 0.05 which were analyzed by one-way ANOVA.*

### Soil Fungal Community Composition

**FIGURE 1:**
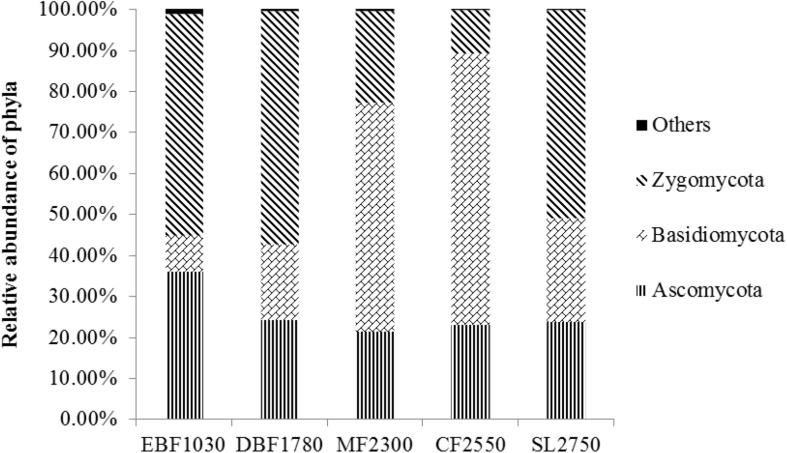
Relative abundance of major phyla at the five forest sites. The relative abundance of Chytridiomycota and Glomeromycota were less than 1%. 0.53% sequencing reads belonged to unclassified or unidentified fungal OTUs and were classified as Others.

A total of 7,897 fungal OTUs were detected and assigned to five known fungal phyla (Ascomycota, Basidiomycota, Zygomycota, Chytridiomycota, Glomeromycota) and 393 known genera. The dominant soil fungal phyla differed among forest types (Figure 1). Zygomycota was the dominant phylum in EBF1030 (53.79%), DBF1780 (56.80%) and SL2750 (50.52%), while Basidiomycota was dominant in MF2300 (55.57%) and CF2550 (65.99%). At the genus level, *Mortierella*, *Cryptococcus*, and *Pseudogymnoascus* were the most abundant groups among Zygomycota, Basidiomycota, and Ascomycota, respectively (Supplementary Table S1). At the order level, Mortierellales and Mucorales (belonging to Incertae_sedis_10) and Filobasidiales (Tremellomycetes) were the most abundant groups (Supplementary Table S2). At the class level, Incertae_sedis_10, Agaricomycetes and Tremellomycetes were the most abundant groups corresponding to the order level (Supplementary Table S3). Detrended correspondence analysis was performed to analyze the soil fungal community structure among all five sampling sites. Results showed that collected replicates from each site were closely grouped (Supplementary Figure S2). To further examine differences, the Anosim non-parametric multivariate dissimilarity test indicated that the soil fungal community structure differed significantly among the five sites (*P* < 0.05) (Supplementary Table S4).

**FIGURE 2:**
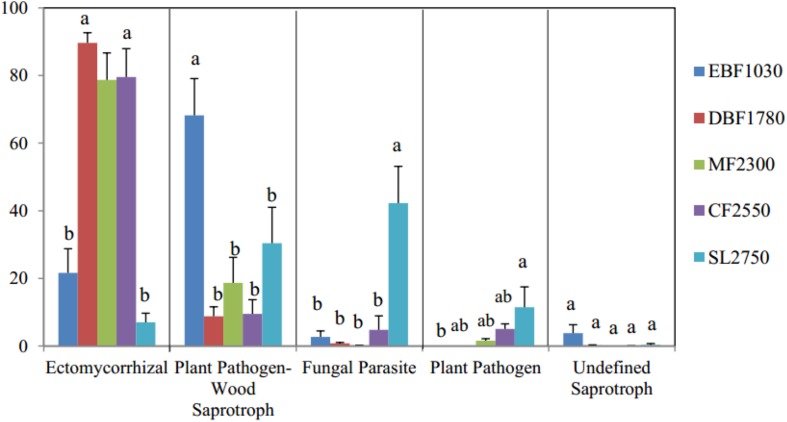
Function predictions of soil fungal communities using FUNGuild method. The *y* axis represents the relative OTUs abundance and different letters above the columns mean significant difference among samples (*P* < 0.05).

OTUs with highly probable functional assignments from FUNGuild (1284 OTUs, or 16.3% of total OTUs) were used for functional analysis. Six main trophic modes (Pathotroph, Saprotroph, Symbiotroph, Saprotroph-Symbiotroph, Pathotroph-Symbiotroph and Pathotroph- Saprotroph) including 10 functional guilds were detected by FUNGuild. The four most abundant guilds (Ectomycorrhizal is 56.71% on average, Plant Pathogen-Wood Saprotroph is 25.43% on average, Fungal Parasite is 10.44% on average, and Plant Pathogen is 3.77% on average) and the undefined saprotroph (0.76% on average) were selected to determine the relationship between the fungal functional assembly (Figure 2). Pearson correlation showed that the Shannon diversity index had a significant correlation with relative abundance percentage of ectomycorrhizal, fungal parasite and plant pathogen.

Overall, results indicate that dominant phyla among soil fungal communities differed significantly among forest types, with ectomycorrhizal being most abundant among fungal functional gulid with a highly probable confidence ranking.

### Soil Fungal Distribution Patterns Along Elevational Gradients

The Shannon index ranged from 3.64 ± 0.26 to 2.51 ± 0.14, and the richness index ranged from 752.20 ± 77.39 to 327.30 ± 30.62 (Table 1). Soil fungal α-diversity analysis showed that the Shannon index and richness index significantly decreased (*P* < 0.01) as elevation increased (Supplementary Figure S1B). Moreover, the fungal phyla showed varying distribution patterns with increasing elevation. For example, the relative abundance of Zygomycota decreased significantly with increasing elevation, while Basidiomycota increased and Ascomycota showed no difference.

**FIGURE 3:**
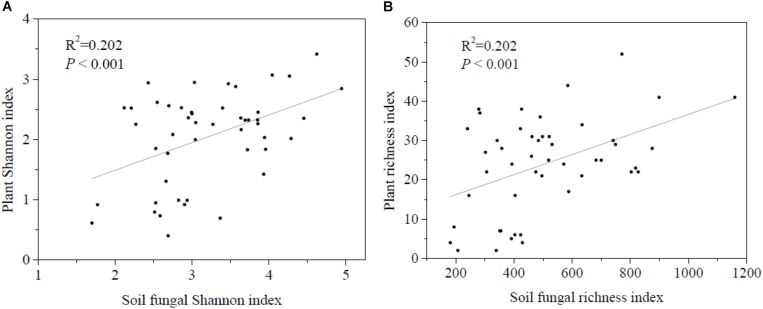
Relationships between soil fungal α-diversity and plant α-diversity. **(A)** Shannon diversity of soil fungi and plant and **(B)** richness diversity of soil fungal and plant.

**FIGURE 4:**
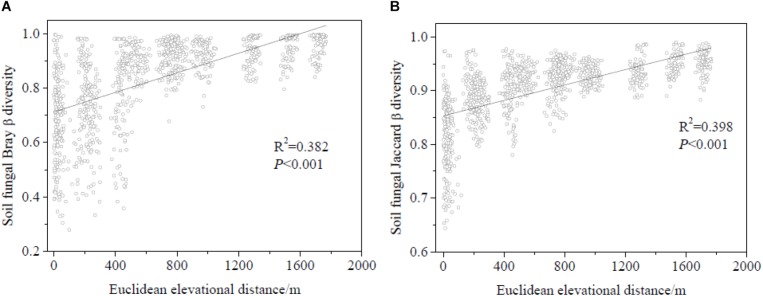
Relationship between soil fungal β-diversity and elevational distance (based on Euclidean distance). **(A)** Soil fungal β-diversity was calculated by Bray-Curtis and **(B)** soil fungal β-diversity was calculated by Jaccard.

Regression analysis revealed a significant positive correlation between plant and soil fungal Shannon diversity and richness (Figure 3). Soil fungal β-diversity analysis revealed that the pairwise soil fungal community compositional dissimilarity among sites increased as elevational distance increased (Figure 4).

### Soil Fungal Community Ecological Assembly Process

The quantified community assembly processes analysis showed that 26.67–93.00% of turnover in community composition was due to variable selection, 0–68.89% was due to dispersal limitation acting alongside drift, 0–73.33% due to drift acting alone, and 0–2.22% was due to homogenizing selection (Table 2). It showed that the relative contribution of stochastic processes (37.78–73.33%) was higher than that of deterministic processes (26.67–62.22%) within soil fungal groups of each forest type, and deterministic processes were higher (35.00–93.00%) than those of stochastic processes (7.00–65.00%) in different forest types.

**TABLE 2:** The contribution rates of the dispersal limitation, drift, and selection processes to structuring the soil fungal community composition along the elevational gradient.

**Study sites**	**Stochastic processes**			**Deterministic processes**		
	**Dispersal limitation**	**Drift**	**Total**	**Variable selection**	**Homogenizing selection**	**Total**
SL2750	2.22%	53.33%	55.55%	44.45%	0.00	44.45%
CF2550	68.89%	0.00	68.89%	31.11%	0.00	31.11%
MF2300	0.00	73.33%	73.33%	26.67%	0.00	26.67%
DBF1780	2.22%	35.56%	37.78%	60.00%	2.22%	62.22%
EBF1030	21.43%	46.43%	67.86%	32.14%	0.00	32.14%
SL2750-CF2550	1.00%	6.00%	7.00%	93.00%	0.00	93.00%
SL2750-MF2300	3.00%	14.00%	17.00%	83.00%	0.00	83.00%
SL2750-DBF1780	38.00%	14.00%	52.00%	48.00%	0.00	48.00%
SL2750-EBF1030	50.00%	15.00%	65.00%	35.00%	0.00	35.00%
CF2550-MF2300	0.00	46.00%	46.00%	54.00%	0.00	54.00%
CF2550-DBF1780	32.00%	5.00%	37.00%	70.00%	0.00	70.00%
CF2550-EBF1030	25.00%	5.00%	30.00%	70.00%	0.00	70.00%
MF2300-DBF1780	22.00%	16.00%	38.00%	61.00%	1.00%	62.00%
MF2300-EBF1030	26.25%	5.00%	31.25%	68.75%	0.00	68.75%
DBF1780-EBF1030	58.75%	1.25%	60.00%	40.00%	0.00	40.00%

Within soil fungal groups of each forest type, drift and dispersal limitation accounted for a large percentage of community compositional turnover rates in four out of five sites, including MF2300 (73.33%), CF2550 (68.89%), EBF 1030 (67.86%) and SL2750 (55.56%). Drift only processes accounted for the highest contribution rate (41.73% on average), indicating it may play vital roles in stochastic processes within soil fungal groups. However, among different forest types, the variable selection process dominated the assembly mechanism, accounting for 54.00–93.00% of the turnover process between CF2550 and MF2300, DBF1780, EBF1030, and SL2750, and 54.00–83.00% of the turnover process between MF2300 and SL2750, CF2550, DBF1780, and EBF1030. What’s more, high rates of dispersal limitation (58.75%) were found in turnover between lower elevational soils in EBF1030 and DBF1780, while only 1% was found between the two higher elevational soils in SF2750 and CF2550. Therefore, variable selection and high levels of dispersal limitation processes may have large impacts on community composition among plant types.

### Linkage Between Soil Fungal Community and Environmental Factors

The Pearson correlation (Table 3) showed that soil fungal α-diversity indices were significantly related to pH, soil moisture, MAP, MAT and plant α-diversity. The partial Mantel test showed that MAT and MAP played important roles in the soil fungal and plant beta diversity (Table 4), while the partial Mantel test showed that plant β-diversity was significantly (*P* < 0.001) correlated with soil fungal β-diversity. These results indicate that soil fungal communities were greatly affected by climate factors along the elevational gradient.

**TABLE 3:** Pearson correlations between soil fungal ITS diversity and several site parameters.

**Parameters**	**Soil fungal ITS**		**Soil fungal ITS**	
	**Shannon index**		**richness index**	
	***r***	***P***	***r***	***P***
pH	0.416	0.003	0.309	0.032
Soil moisture	0.535	< 0.001	0.612	< 0.001
Plant Shannon index	0.449	0.001	0.375	0.009
Plant richness index	0.518	< 0.001	0.449	0.001
Annual mean precipitation	–0.501	< 0.001	–0.426	0.003
Annual mean temperature	0.502	< 0.001	0.438	0.002
Soil organic carbon	–0.274	0.059	–0.384	0.007

**TABLE 4:** Partial Mantel test between β-diversity of plant and soil fungi and environmental properties.

**Environmental factors**	**Plant β-diversity**		**Soil fungi β-diversity**	
	***r***	***P***	***r***	***P***
Soil organic carbon	0.134	< 0.001	0.197	0.002
Available nitrogen	0.102	0.015	0.038	0.252
Available phosphorus	0.024	0.399	–0.055	0.674
Soil pH	0.211	< 0.001	0.309	< 0.001
Soil moisture	0.270	< 0.001	0.121	0.025
Mean annual precipitation	0.405	< 0.001	0.515	< 0.001
Mean annual temperature	0.391	< 0.001	0.521	< 0.001

## Discussion

In this study, the forest soil fungal community composition of Shennongjia Mountain was primarily composed of Zygomycota, Basidiomycota, Ascomycota, Chytridiomycota, and Glomeromycota. This composition is similar to results observed in other studies (Siles and Margesin, 2016; Liu et al., 2018). Previous work has suggested that Ascomycota (Geml et al., 2014; Yang et al., 2017) or Basidiomycota (Liu et al., 2018) were the dominant phyla in other soil types. However, our study showed that the dominant phylum was inconsistent among plant types. Zygomycota was the dominant phylum in EBF1030, DBF1780 and SL2750 (Wallenstein et al., 2007; Chu et al., 2011; Chen et al., 2019). Chen et al. (2019) found that Zygomycota accounted for 45% of the phyla in primary stands in tropical rainforests, but decreased to 25% in select cut stands and to 26% in clear cut stands. These findings indicate soil fungal communities are influenced by vegetation type, evolutionary history and environmental factors. For example, research has shown that soil fungal communities are dependent on vegetation type (Wu et al., 2013; Shi et al., 2014). Ascomycota and Basidiomycota have high relative abundance in cooler, arid environments because of their evolutionary histories (Treseder et al., 2014; Tian et al., 2017), while Zygomycota dominate shrub soils (Wallenstein et al., 2007; Chu et al., 2011).

The software FUNGuild is valuable due to its ability to discern the functional roles of fungi (Wei et al., 2018). Microbes can be classified as slow-growing oligotrophs or fast-growing copiotrophs according to their life history strategies (Fierer et al., 2007). Within the oligotrophic fungal group, the relative abundance of Basidiomycota high significantly (*P* < 0.001). Most of the ectomycorrhizal OTUs were classified to Basidiomycota. Ectomycorrhizal (symbiotroph) can receive nutrients by exchanging resources with host cells (Nguyen et al., 2016). This is consistent with our finding that ectomycorrhizal was significantly positively related to plant diversity in our samples, improving abundance of each with the assistance of the other. Past studies have suggested that the increase in abundance of Basidiomycota along elevational gradients may be related to their ability to decompose the recalcitrant lignocellulose found in coniferous litter (Lundell et al., 2010; Siles and Margesin, 2016; Liu et al., 2018). The growth of Basidiomycota, including its most abundant genus *Cryptococcus* (belonging to Filobasidiales), exhibited this trend in past research, indicating that *Cryptococcus* may prefer higher elevations. A similar result was found for Filobasidiales, which was dominant between 1500 and 3000 masl (Geml et al., 2014). Fungal parasites and plant pathogens both showed a significant negative correlation with plant diversity in our study, which is not unsurprising as they are pathotrophs that receive nutrients by harming host cells (Nguyen et al., 2016). This relationship may be an important contributor to the decline of plant diversity.

Distinct soil fungal elevational distribution patterns may be due to processes brought together by a combination of factors. The elevational distribution pattern of soil fungi in this study was slightly different from that of soil bacteria in Shennongjia Mountain (Zhang et al., 2015), where it was reported that the Shannon and Simpson indices of soil bacteria decreased from 1050–2550 masl, then increased at 2750 masl. This inconsistent pattern is in agreement with results reported by Liu et al. (2018), who also found different elevational patterns in soil fungal and bacterial diversity on Mount Nadu (Liu et al., 2016, 2018). Inconsistencies are likely shaped by different processes (Liu et al., 2018). It has been previously suggested that diversity assemblage processes of diversity of soil fungi and bacteria from the surface and subsurface may differ (Yang et al., 2017). Although the soil fungi (this study) and bacterial (Zhang et al., 2015) communities were significantly related to plant diversity, soil fungi form more mutualistic symbioses with plant species than that of bacteria, suggesting adequate nutrient cycling that ultimately works to enhance fungal diversity.

The results of this study showed that drift processes played an important role in shaping fungal composition within the same plant type, likely due to decreasing habitat heterogeneity within the same plant type and diminished effects of environmental fluctuations (Bahram et al., 2016). Once interstitial pore spaces are filled with water after rain, they can be easily interconnected on a small local scale, increasing compositional stochasticity through ecological drift and weakening niche selection (Valverde et al., 2014). Further, the spreading mycelium of soil fungi can reach up to 100 m in extreme cases, facilitating dispersal within the same site (Morrison-Whittle and Goddard, 2015; Bahram et al., 2016). The dispersal limitation and drift were substantial among the lower elevation sites, leading to homogenization among communities. This may be linked to greater human disturbance at lower elevations. High levels of dispersal limitation were found in turnover between lower elevational soils EBF1030 and DBF1780 and others in our study. High levels of dispersal limitation could lead to spatial turnover in microbial communities (Martiny et al., 2011; Stegen et al., 2013), in contrast, this dropped (1%) only when comparing the two higher elevation sites, suggesting that high level dispersal limitation are important in shaping community composition when environmental conditions are less stringent. The null model demonstrated that selection processes made a greater contribution to the assembly process of soil fungal community patterns between plant types. Selection induces differential survival and reproductive success across individuals and species, then constrains and differentiates microbial community compositions among locations (Hanson et al., 2012; Stegen et al., 2013).

Selection is caused by biotic pressures such as competition, predation, and mutualism (Hanson et al., 2012), as well as abiotic pressures such as environmental, physical, and chemical properties (Fierer and Jackson, 2006). Elevational gradients can have highly differing ecological conditions (Liu et al., 2018), resulting in the environmental filtering and niche segregation of soil microbes, as well as the heterogeneous habitats of soil fungal communities between plant types (Bahram et al., 2016; Moroenyane et al., 2016). Morrison-Whittle and Goddard (2015) found that selection was the dominant process shaping fungal communities over large scales and suggested that differences between soil communities across locations may originate as selective effects. Other studies have demonstrated that stochastic and deterministic processes dominated the surface and subsurface soil fungal communities in high elevation desert environments (Yang et al., 2017), and that stochastic and deterministic processes dominated the wet soil (1–10 m from the waterline) and dry soil (20 m from the waterline) bacteria (Valverde et al., 2014). These findings help support the prediction that ecological processes depend on selected scales or niches.

In summary, soil fungal diversity in different forest types showed marked divergence and decreased significantly (*P* < 0.01) with increasing elevation. Both deterministic and stochastic processes structured the soil fungal communities, with deterministic processes being dominant among different plant types. Climate parameters (MAP and MAT) appear to be key environmental factors shaping soil fungal community structure along elevational gradients. Overall, the results of this study provide new insight into soil fungal distribution and community assembly processes in natural ecosystems.

## Data Availability Statement

The sequencing datasets analyzed during the current study are available in the GenBank database with accession number of SRP222129.

## Author Contributions

YZ developed and framed the research questions. YS, WC, LY, and QL finished the plant survey and collected the data used in this analysis. YS led the data analyses and wrote the first draft of the manuscript. All authors contributed substantially to revisions.

## Conflict of Interest

The authors declare that the research was conducted in the absence of any commercial or financial relationships that could be construed as a potential conflict of interest.

## Footnotes

1 http://www.worldclim.org

2 http://mem.rcees.ac.cn:8080

3 http://ieg.ou.edu/
