# Corrigendum: Forest Soil Fungal Community Elevational Distribution Pattern and Their Ecological Assembly Processes

**DOI:** 10.3389/fmicb.2019.02802

**Published:** 2019-12-05

**Authors:** Yuyu Sheng, Wei Cong, Linsen Yang, Qiang Liu, Yuguang Zhang

**Affiliations:** ^1^Research Institute of Forest Ecology, Environment and Protection, Chinese Academy of Forestry, and the Key Laboratory of Biological Conservation of National Forestry and Grassland Administration, Beijing, China; ^2^Shennongjia National Park Administration, and Hubei Provincial Key Laboratory on Conservation Biology of the Shennongjia Golden Monkey, Shennongjia, China

**Keywords:** soil fungal diversity, community assembly processes, elevational gradient, environmental factors, Illumina MiSeq sequencing

In the original article, there was a mistake in the legend for **Figure 2** as published. The sentence “Relationships between (a) α-diversity of plant and soil fungi and (b) soil fungal α-diversity and elevational gradient” in the legend should be deleted. The correct legend appears below.

**Figure 2 |** Function predictions of soil fungal communities using FUNGuild method. The *y* axis represents the relative OTUs abundance and different letters above the columns mean significant difference among samples (*P* < 0 05).

The authors apologize for this error and state that this does not change the scientific conclusions of the article in any way. The original article has been updated.

