# Supplementary material for: Forest Soil Fungal Community Elevational Distribution Pattern and Their Ecological Assembly Processes
Source: Front Microbiol. 2019 Oct 4;10:2226. doi: 10.3389/fmicb.2019.02226 (PMC6787267; doi:10.3389/fmicb.2019.02226)
Supplement: Supplementary file 1 [file Table_1.DOCX]

**Supporting Materials**

Table S1 Relative abundance of soil fungal genera with ≥ 1% in at least one site

| Phylum | Genus | EBF1030 | DBF1780 | MF2300 | CF2550 | SL2750 |
| --- | --- | --- | --- | --- | --- | --- |
| Ascomycota | *Acicuseptoria* | 0.001±0.00b | 0.001±0.00b | 0.13±0.03b | 1.11±0.42a | 0.11±0.07b |
|  | *Davidiella* | 1.07±0.49a | 0.10±0.03b | 0.93±0.24ab | 0.21±0.05ab | 0.13±0.04b |
|  | *Hypocrea* | 0.06±0.03a | 0.05±0.01a | 0.01±0.00a | 0.76±0.61a | 1.09±0.58a |
|  | *Neurospora* | 0.02±0.00a | 0.01±0.00a | 0.01±0.00a | 0.18±0.18a | 6.67±6.15a |
|  | *Phoma* | 7.37±2.63a | 0.63±0.15b | 1.11±0.35b | 2.70±1.63ab | 0.81±0.45a |
|  | *Pseudogymnoascus* | 3.28±1.59a | 10.99±4.48a | 15.07±3.02a | 15.03±2.49a | 9.24±2.65a |
|  | *Trichoderma* | 4.08±2.42a | 0.71±0.25ab | 0.02±0.01b | 0.01±0.00b | 0.15±0.13b |
| Basidiomycota | *Cortinarius* | 0.05±0.04a | 2.67±1.44a | 0.49±0.29a | 2.73±1.05a | 0.01±0.00a |
|  | *Cryptococcus* | 0.38±0.16b | 1.36±0.38b | 24.80±4.81a | 29.01±5.95a | 22.63±7.77a |
|  | *Hygrophorus* | 0.01±0.00a | 0.04±0.02a | 2.41±2.39a | 4.94±4.89a | 0.002±0.00a |
|  | *Inocybe* | 0.04±0.03b | 0.10±0.06b | 4.44±2.10a | 2.35±0.70ab | 0.003±0.00b |
|  | *Russula* | 0.58±0.37ab | 3.99±1.60ab | 0.49±0.16ab | 4.34±1.61a | 0.03±0.02b |
|  | *Sebacina* | 0.32±0.19ab | 0.43±0.08b | 2.58±0.81ab | 10.30±5.21a | 0.02±0.01b |
| Zygomycota | *Mortierella* | 44.07±10.53a | 7.66±1.69b | 12.95±3.57b | 9.78±4.14b | 31.58±8.19ab |
|  | *Mucor* | 0.63±0.32a | 1.28±0.71a | 1.25±0.48a | 0.67±0.35a | 5.85±3.90a |
|  | *Umbelopsis* | 2.25±1.04b | 47.72±7.51a | 8.10±7.18b | 0.10±0.05b | 13.06±5.71b |

Table S2 Relative abundance of soil fungal order with ≥ 5% in at least one site

| Phylum | Class | Order | EBF1030 | DBF1780 | MF2300 | CF2550 | SL2750 |
| --- | --- | --- | --- | --- | --- | --- | --- |
| Ascomycota | Dothideomycetes | Incertae_sedis_8 | 3.29±1.59a | 11.00±4.48a | 15.08±3.02a | 15.28±2.52a | 9.73±2.66a |
|  |  | Pleosporales | 13.57±4.69a | 1.33±0.26b | 1.54±0.35b | 4.01±1.74b | 1.11±0.49b |
|  | Sordariomycetes | Hypocreales | 14.36±9.22a | 8.23±1.76a | 0.16±0.04a | 1.08±0.73a | 2.55±0.95a |
|  |  | Sordariales | 0.11±0.07a | 0.09±0.03a | 0.02±0.01a | 0.94±0.93a | 6.73±6.19a |
| Basidiomycota | Agaricomycetes | Agaricales | 3.75±2.38bc | 8.75±2.42abc | 20.23±4.15a | 15.32±4.51ab | 0.06±0.01c |
|  |  | Sebacinales | 0.69±0.27b | 1.14±0.39b | 4.30±1.05ab | 11.80±5.19a | 0.06±0.02b |
|  |  | Russulales | 0.74±0.42ab | 4.84±1.81a | 0.77±0.19ab | 4.57±1.62a | 0.03±0.02b |
|  | Tremellomycetes | Filobasidiales | 0.38±0.16b | 1.36±0.38b | 24.80±4.81a | 29.01±5.95a | 22.63±7.77a |
| Zygomycota | Incertae_sedis_10 | Mortierellales | 50.90±12.93a | 7.67±1.69b | 13.08±3.62b | 9.82±4.14b | 31.65±8.19ab |
|  |  | Mucorales | 2.90±1.30b | 49.13±7.36a | 9.35±7.06b | 0.78±0.39b | 18.90±8.51b |

Table S3 Relative abundance of soil fungal class with ≥ 5% in at least one site

| Phylum | Class | EBF1030 | DBF1780 | MF2300 | CF2550 | SL2750 |
| --- | --- | --- | --- | --- | --- | --- |
| Ascomycota | Dothideomycetes | 18.09±6.44a | 12.61±4.50a | 17.85±3.17a | 19.57±3.09a | 11.13±2.71a |
|  | Sordariomycetes | 15.20±9.30a | 9.35±1.95a | 0.39±0.09a | 2.11±1.69a | 9.56±6.59a |
| Basidiomycota | Agaricomycetes | 7.03±2.87c | 16.04±3.66bc | 29.87±5.92ab | 36.12±5.49a | 0.25±0.06c |
|  | Tremellomycetes | 1.05±0.33a | 1.97±0.45a | 25.40±4.85b | 29.54±5.97b | 24.23±8.45b |
| Zygomycota | Incertae_sedis_10 | 53.79±11.89ab | 56.8±6.55a | 22.43±6.58bc | 10.6±4.40c | 50.52±10.49ab |

Data presented above are percentage of each genus (a) which had an relative abundance of ≥ 1%, and each order (b) or class (c) of 5% at least one site. Unclassified genera in each phylum were not shown. Data ware presented in mean value ± standard error, and different lowercase letters within the same row indicate significant difference at *P* *<* 0.05 which were analyzed by one-way ANOVA. EBF1030 (evergreen broadleaved forest at the altitude of 1030 masl), DBF1780 (deciduous broadleaved forest at the altitude of 1780 masl), MF2300 (mixed coniferous and deciduous broadleaved forest at the altitude of 2300 masl), CF2550 (coniferous forest at the altitude of 2550 masl) SL2750 (shrubland at the altitude of 2750 masl).

Table S4 Anosim dissimilarity test of soil fungal community in this study

| Sample name | Bray-Curtis | | Jaccard | |
| --- | --- | --- | --- | --- |
|  | R value | *P* | R value | *P* |
| EBF1030-DBF1780 | 0.822 | 0.001 | 0.772 | 0.001 |
| EBF1030-MF2300 | 0.853 | 0.001 | 0.872 | 0.001 |
| EBF1030-CF2550 | 0.867 | 0.001 | 0.903 | 0.001 |
| EBF1030-SL2750 | 0.801 | 0.001 | 0.890 | 0.001 |
| DBF1780-MF2300 | 0.938 | 0.001 | 0.988 | 0.001 |
| DBF1780-CF2550 | 0.950 | 0.001 | 0.990 | 0.001 |
| DBF1780-SL2750 | 0.826 | 0.001 | 0.966 | 0.001 |
| MF2300-CF2550 | 0.125 | 0.027 | 0.571 | 0.001 |
| MF2300-SL2750 | 0.237 | 0.001 | 0.648 | 0.001 |
| CF2550-SL2750 | 0.310 | 0.001 | 0.758 | 0.001 |


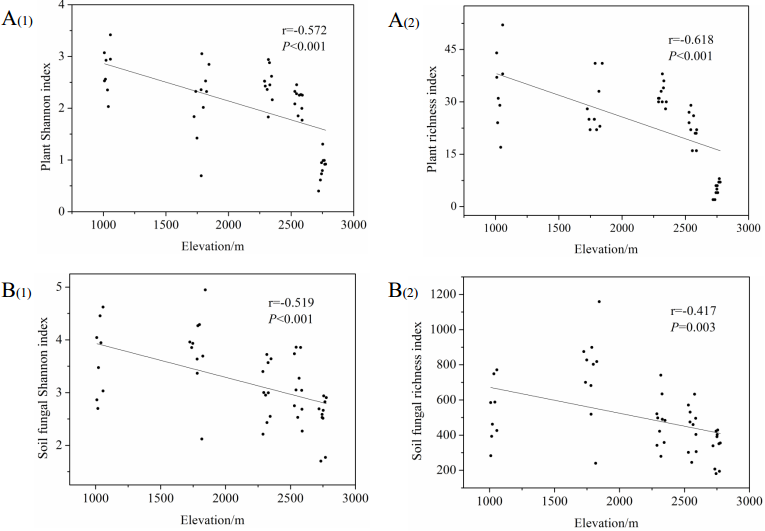


Fig S1 Pearson correlation tests of relationships between (A) plant α − diversity and elevational gradient, (B) soil fungal α − diversity and elevational gradient.





Fig S2 Detrended correspondence analysis of soil fungal community at the five forest sites.

EBF1030 (evergreen broadleaved forest at the altitude of 1030 masl), DBF1780 (deciduous broadleaved forest at the altitude of 1780 masl), MF2300 (mixed coniferous and deciduous broadleaved forest at the altitude of 2300 masl), CF2550 (coniferous forest at the altitude of 2550 masl) and SL2750 (shrubland at the altitude of 2750 masl)
